# Association Mapping of Seed Oil and Protein Content in *Sesamum indicum* L. Using SSR Markers

**DOI:** 10.1371/journal.pone.0105757

**Published:** 2014-08-25

**Authors:** Chun Li, Hongmei Miao, Libin Wei, Tide Zhang, Xiuhua Han, Haiyang Zhang

**Affiliations:** Henan Sesame Research Center, Henan Academy of Agricultural Sciences, Zhengzhou, Henan, People's Republic of China; Nanjing Agricultural University, China

## Abstract

Sesame is an important oil crop for the high oil content and quality. The seed oil and protein contents are two important traits in sesame. To identify the molecular markers associated with the seed oil and protein contents in sesame, we systematically performed the association mapping among 369 worldwide germplasm accessions under 5 environments using 112 polymorphic SSR markers. The general linear model (GLM) was applied with the criteria of log*P*≥3.0 and high stability under all 5 environments. Among the 369 sesame accessions, the oil content ranged from 27.89%–58.73% and the protein content ranged from 16.72%–27.79%. A significant negative correlation of the oil content with the protein content was found in the population. A total of 19 markers for oil content were detected with a R^2^ value range from 4% to 29%; 24 markers for protein content were detected with a R^2^ value range from 3% to 29%, of which 19 markers were associated with both traits. Moreover, partial markers were confirmed using mixed linear model (MLM) method, which suggested that the oil and protein contents are controlled mostly by major genes. Allele effect analysis showed that the allele associated with high oil content was always associated with low protein content, and vice versa. Of the 19 markers associated with oil content, 17 presented near the locations of the plant lipid pathway genes and 2 were located just next to a fatty acid elongation gene and a gene encoding Stearoyl-ACP Desaturase, respectively. The findings provided a valuable foundation for oil synthesis gene identification and molecular marker assistant selection (MAS) breeding in sesame.

## Introduction

Sesame (*Sesamum indicum* L.) is an ancient and important oilseed crop and is cultivated mainly in the tropical and subtropical regions of Asia, Africa and Southern America. The harvested area of world sesame reaches to 7.3×10^7^ hm^2^, and the total product per year is roughly 3.7×10^7^ ton from 2001 to 2010 (FAO data). Compared with other main oil crops, e.g., soybean (18% of average oil content) [1], oilseed rape (41%) [2], sunflower (40–44%) [3] and peanut (51%) [4], sesame is one of the few oil crops with the highest oil content and quality. Sesame seeds contain 55–58% oil and almost 18% proteins. Among the fatty acid compositions in sesame seeds, oleic acid (18∶1) (39.6%) and linoleic acid (18∶2) (46.0%) are the two main components with the ideal ratio of almost 1∶1 [5], [6]. Apart from the seed yield, the content of seed storage oil and proteins is the highlight agronomic trait in sesame breeding [7].

In the past two decades, in order to clarify the high quality of sesame oil and protein, many researchers focused on exploring seed development and fatty acid and storage protein synthesis processes, as well as identifying the lipid synthesis related genes and molecular markers in sesame [8]–[12]. Of all the three available cDNA libraries, two libraries are constructed for seed development analysis [13], [14]. However, oil and protein contents are complex quantitative traits and always are affected by genotype and environment [15]. At present, the mechanism of high oil content and quality in sesame seeds is still unclear. No loci of oil and protein content traits have ever been found in the sesame linkage maps. Even though Wei et al. [12] performed the association analysis of seed oil and protein content and fatty acid composition within 216 Chinese sesame accessions using 79 molecular primer pairs (including SSRs, SRAPs and AFLPs), only one association marker (M15E10-3) was identified under two environments. Therefore, in order to precisely detect the genes or markers associated with oil and protein content traits and to improve the sesame breeding, more efficient markers and germplasm resources with larger phenotypic variation need to be applied [16], [17].

Currently, linkage analysis (QTL mapping) and association mapping are two main and common analysis tools for dissecting complex phenotypic variation. Compared with the traditional linkage analysis based on mapping populations, association mapping offers higher precision for locating QTLs and selecting molecular markers [16], [18]. Till now, association mapping has been extensively used for analyzing important agronomic and quantity traits in wheat, maize, cotton, oilseed rape and other crops [18]–[22]. In the past several years, vast simple sequence repeat (SSR) or microsatellite markers with the high polymorphism are developed in sesame [7], [23], [24]. Accordingly, the association mapping is getting reliable and powerful for detecting the genes or markers associated with key traits and improving the molecular marker-assisted selection (MAS) in sesame breeding programs.

The objectives of this study are: (1) to perform the association mapping of seed oil content (OC) and protein content (PC) traits in worldwide sesame accessions using the GLM and MLM models, (2) to reflect the characteristics of sesame oil and protein contents under various environments, and (3) to determine the key SSR markers associated with seed quality. In this report, a natural population covering 369 worldwide accessions from China and other 15 countries and 112 pairs of polymorphic SSR markers were applied. The results give a foundation for investigating the seed development-related genes and seed quality in sesame.

## Results

### Seed oil and protein content variation in the natural population

Both the seed oil content (OC) and the protein content (PC) are often influenced by environment. To decrease the environmental effect, we collected the phenotypic data of the 369 sesame accessions under 5 environments of Pingyu and Yuanyang locations in 2011, and Pingyu, Yuanyang and Xinyang locations in 2012, The descriptive parameters under each environment were calculated (Table 1). The results showed that the OC and PC significantly varied among the 369 accessions. In total, the OC of the natural population ranged from 27.89%–58.73%, with an average of 49.59%–53.14%; meanwhile, the PC ranged from 16.72%–27.79% with an average of 20.28%–22.51%. All the datasets showed a normal or nearly normal distribution. To determine the heritability of the phenotypes, we performed the variance analysis of oil and protein contents (Table 2). Results indicated that the OC and PC traits were significantly influenced by genotype and environments (i.e., year and location). No significant interactions between variety and environmental factors (year and location) were detected. Moreover, the OC and PC traits presented the significant negative correlation under the 5 environments, as the correlation coefficient (*r*) between OC and PC varied from −0.66∼−0.72 (*P*<0.01) in 2011 and from −0.52∼−0.74 (*P*<0.01) in 2012, respectively (Data not listed).

**Table 1:** Phenotypic variation of the seed oil and protein contents in the 369 accessions under 5 environments.

Trait	Year	Place	Min. (%)	Max. (%)	Mean (%)	Std. ev.	Skewness	Kurtosis
OC	2011	Pingyu	27.89	55.03	49.59	4.61	−2.19	1.69
	2011	Yuanyang	34.28	57.53	51.94	3.72	−2.26	2.85
	2012	Pingyu	31.95	54.80	49.82	3.43	−2.54	5.13
	2012	Yuanyang	30.77	57.88	52.23	4.22	−2.53	3.98
	2012	Xinyang	32.74	58.73	53.14	4.59	−2.32	2.67
PC	2011	Pingyu	18.74	26.49	21.65	1.30	0.90	1.28
	2011	Yuanyang	17.60	25.10	20.28	1.16	0.97	1.55
	2012	Pingyu	20.22	27.79	22.51	1.04	0.78	1.68
	2012	Yuanyang	16.72	27.44	20.94	1.25	1.05	5.27
	2012	Xinyang	17.24	26.84	20.59	1.57	0.85	1.30

**Table 2:** Analysis of Variance of the oil and protein contents in the population under 5 environments.

Source of variation	DF	OC		PC	
		MS	F value	MS	F value
Year	1	27.33	7.46**	211.90	412.83**
Location	2	1564.58	427.02**	551.56	1074.55**
Variety	368	62.78	17.13**	5.23	10.19**
Year×Variety	368	3.19	0.87	0.55	1.07
Location×Variety	736	2.94	0.80	0.65	1.27
Residual	367	3.66		0.51	

** The significance at *P*<0.01.

DF denotes degree of freedom; MS denotes mean square.

### Linkage disequilibrium

**Figure 1:**
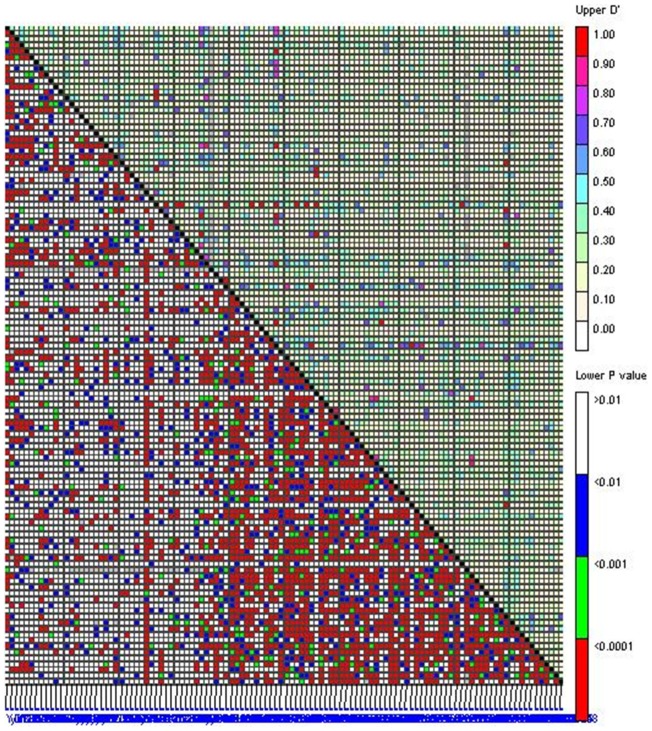
Disequilibrium matrix of 112 SSR polymorphic sites with both the X-axis and Y-axis. The matrix is divided into two regions by the diagonal line. The upper right region indicates the D′ value of each SSR couples, and the corresponding blocks in the lower left region indicates the significance of D′. Various value intervals of the D′ or P values are shown in different colors according to the right color columns.

Linkage disequilibrium (LD) refers to the non-random association of alleles between the genetic loci. A total of 112 SSR markers were used for estimating the LD level among the 369 sesame germplasm accessions (Table S2). These SSR markers distributed in 33 contigs/scaffolds with a total length of 180.86 Mb, which approximately represented 67 percentage of the assembly genome size (270 Mb) and 50 percentage of the estimated genome size (360Mb). The average SSR density was 1 SSR per 1.6 Mb. To reflect the associations between the polymorphic loci of the 112 SSR markers, LD *P*-values were determined among the 6,216 locus pairs (i.e., 112*(112-1)/2) using Fisher's exact test and two indexes of *D*′ and *r* ^2^ (Figure 1). The average values of *D*′ and *r* ^2^ for the 6,216 pairs were 0.1649 and 0.0173, respectively. Of the 6216 pairs, 2584 pairs (41.57%) showed a significant linkage disequilibrium (*P*<0.01), 363 pairs (5.84%) showed a higher linkage disequilibrium of *D*′>0.5, and 33 pairs (0.5%) gave a *D*′ value of 1.0 (i.e., complete linkage). The data indicated that linkage disequilibrium existed among the sesame accessions.

### SSR marker diversity and population structure

**Figure 2:**
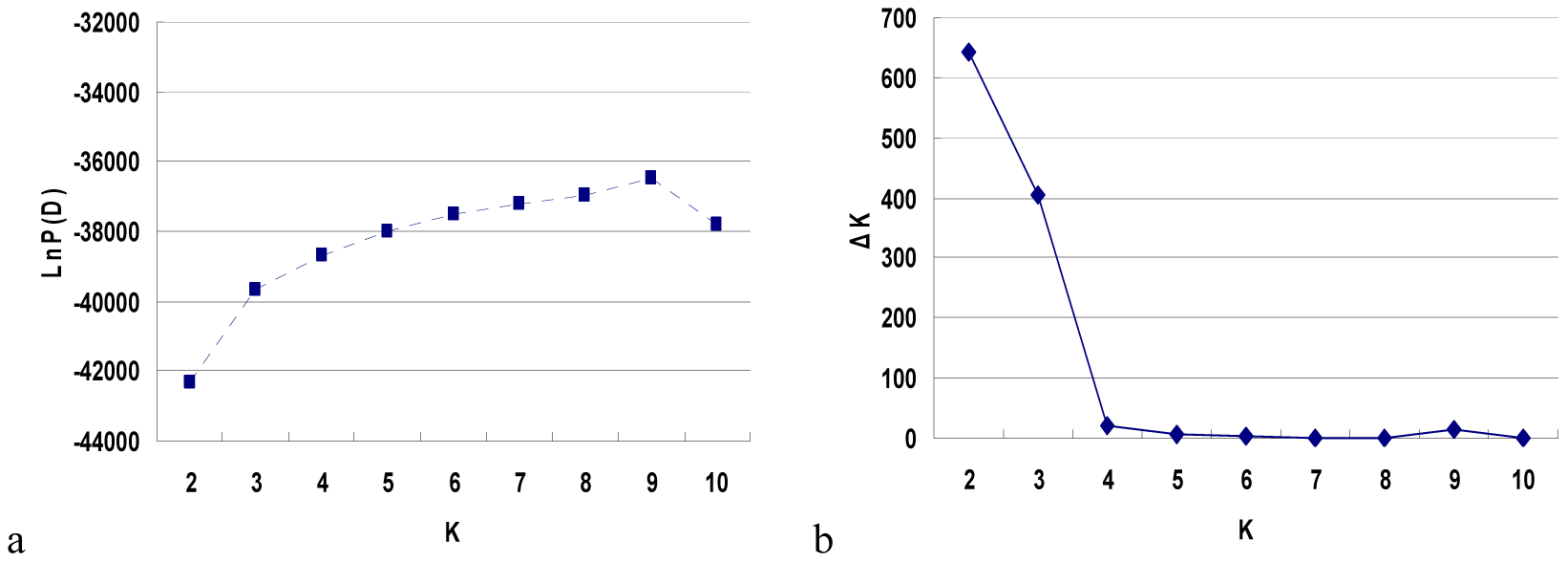
Estimated (a) Ln*P*(D) and (b) Δ*K* values for a given *K*.

**Figure 3:**
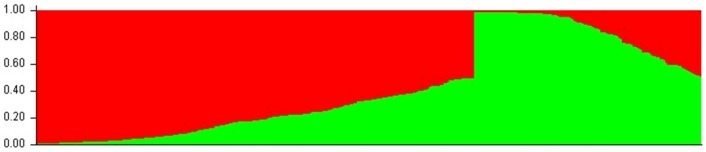
Genetic composition of individuals based on Bayesian posterior probability. According to the Bayesian posterior probability, the natural population of 369 worldwide sesame lines is divided into two groups in green and red colors, respectively.

Before analyzing the association, the polymorphism of the 112 SSR markers within the 369 germplasms was investigated (Table S2). Results showed that the number of alleles ranged from 2–5, with an average of 2.47 per locus. The PIC values of the markers ranged from 0.028 (Hs373) to 0.669 (Y1994) with an average value of 0.302. The percentage of heterozygotes per marker varied from 0.27% (Hs373) to 23.12% (Hs4325), with an average of 10.14%. The data indicated that the natural population had the high heterozygosity and was suitable for association mapping analysis. Subsequently, we estimated the population structure, as admixture of subpopulation could result in LD and produce the false-positive results. The most likely number (*K*) of subgroups in the 369 germplasm accessions was estimated using the 112 SSR markers (Figure 2). As *K* values increased from 1 to 10, the value of Ln*P*(D) elevated directly; meanwhile, the Δ*K* reduced straightly with a clear peak value of 643.5 at *K* = 2. The results indicated that the population was roughly composed of two divergent subgroups according to the Bayesian posterior probability analysis (Figure 3). Of the 369 accessions, 126 accessions were grouped into subgroup 1 (the green ones in Figure 3), 243 accessions into subgroup 2 (the red ones in Figure 3). Most (47 out of 51) of the foreign lines were located in subgroup 2. The two subgroups in the population were considered having the complex ‘admixture’ relationship, and no significant correlation of geographic origin with the subgroups in the Chinese lines were found.

### Marker-trait associations

In this study, the association analysis was performed using general liner model (GLM) method. The stringent criterion of log*P*≥3.0 under 5 environments was used for determining the association significance between the OC and PC traits. For the OC trait, 19 markers were detected and the R^2^ values ranged from 4%–29% (Table 3 & Table S2). According to the primer locations in the sesame genome, 19 markers were mapped in 11 contigs/scaffolds. Of the 19 markers, Hs485 and Hs586 are located in scaffold00001, Hs4381, Hs02 and Hs4082 are located in C01, and Hs4061, Hs345 and Hs19563 are in C04. The distance between the markers that located in the same contig/scaffold was short than 2 Mb (Table S2). For the PC trait, 24 markers were detected with the R^2^ value range of 3%–29% and mapped in 15 contigs/scaffolds (Table 4 & Table S2). Comparison results indicated that 19 of the above 24 markers were associated with OC trait at the same time; the other 5 markers of Hs425 (in C08), Hs560 (in C11), Hs4265 (in C12), and Hs4089 and Hs1514 (in C19) were unique to PC trait. Simultaneously, in order to assay the stability, we performed association mapping using mixed liner model (MLM) method, with the criterion of log*P*≥3.0 under at least 3 environments.

**Table 3:** Association mapping of the OC trait using GLM method under the 5 environments.

Marker	2011						2012								
	Pingyu			Yuanyang			Pingyu			Yuanyang			Xinyang		
	F_maker_	*P*_marker_	R^2^	F_maker_	*P*_marker_	R^2^	F_maker_	*P*_marker_	R^2^	F_maker_	*P*_marker_	R^2^	F_maker_	*P*_marker_	R^2^
Hs345	35.93	3.05E-25	0.28	26.22	4.37E-19	0.22	13.41	3.41E-10	0.13	26.44	3.11E-19	0.22	37.34	4.08E-26	0.29
Hs4381	61.72	8.30E-24	0.25	43.17	1.48E-17	0.19	40.31	1.53E-16	0.18	46.18	1.32E-18	0.20	75.46	3.73E-28	0.29
Hs1956	47.51	7.46E-19	0.22	39.98	2.83E-16	0.19	16.15	2.05E-07	0.09	24.86	8.80E-11	0.13	40.25	2.29E-16	0.19
Hs485	31.50	4.06E-18	0.20	20.63	2.29E-12	0.14	21.10	1.27E-12	0.15	21.48	7.82E-13	0.15	36.07	1.98E-20	0.23
Hs1036	41.73	6.75E-17	0.20	25.69	4.19E-11	0.13	17.87	4.24E-08	0.10	26.00	3.19E-11	0.13	43.84	1.24E-17	0.20
Hs205	17.18	3.11E-15	0.19	10.41	2.42E-09	0.12	10.37	2.68E-09	0.13	12.46	3.65E-11	0.15	16.29	1.75E-14	0.18
Hs4209	14.91	2.67E-13	0.17	8.15	2.64E-07	0.10	9.28	2.55E-08	0.12	12.37	4.41E-11	0.14	23.92	9.21E-21	0.25
Hs672	16.81	1.19E-12	0.16	11.35	1.09E-08	0.11	16.82	1.16E-12	0.16	20.27	4.38E-15	0.18	26.53	2.58E-19	0.22
Hs393	17.90	2.01E-13	0.16	10.10	9.18E-08	0.10	12.19	2.61E-09	0.12	16.74	1.32E-12	0.15	29.18	4.76E-21	0.24
Hs4061	30.93	3.93E-13	0.14	19.32	1.06E-08	0.09	21.42	1.60E-09	0.11	23.28	3.05E-10	0.11	30.72	4.66E-13	0.14
Y2129	22.99	4.73E-10	0.13	13.89	1.64E-06	0.08	15.29	4.57E-07	0.09	24.86	9.25E-11	0.13	30.65	6.69E-13	0.16
Hs618	25.22	5.54E-11	0.12	12.90	3.86E-06	0.06	7.54	6.19E-04	0.04	17.66	4.78E-08	0.09	22.91	4.26E-10	0.11
Hs377	4.14	2.01E-05	0.11	3.16	6.87E-04	0.08	3.92	4.49E-05	0.10	6.26	7.83E-09	0.15	7.09	3.63E-10	0.17
Hs635	22.15	8.38E-10	0.11	17.23	7.11E-08	0.08	10.34	4.30E-05	0.05	18.79	1.71E-08	0.09	31.73	1.98E-13	0.15
Hs02	20.98	2.38E-09	0.10	17.19	7.34E-08	0.08	10.31	4.43E-05	0.05	12.41	6.09E-06	0.06	36.20	4.55E-15	0.16
Hs376	19.25	1.15E-08	0.10	11.49	1.45E-05	0.06	12.53	5.52E-06	0.07	16.27	1.72E-07	0.08	21.26	1.89E-09	0.10
Hs1638	17.45	6.23E-08	0.09	7.29	7.96E-04	0.04	18.27	2.97E-08	0.10	19.25	1.23E-08	0.10	24.86	8.66E-11	0.13
Hs586	16.84	1.01E-07	0.08	13.48	2.25E-06	0.07	7.30	7.82E-04	0.04	10.90	2.52E-05	0.06	20.14	5.06E-09	0.10
Hs4082	8.18	3.36E-04	0.04	7.56	6.08E-04	0.04	18.30	2.69E-08	0.09	15.51	3.44E-07	0.08	20.61	3.33E-09	0.10

The markers associated with the OC trait under 5 environments are listed in the table.

**Table 4:** Association mapping of PC trait using GLM method under the 5 environments.

Marker	2011						2012								
	Pingyu			Yuanyang			Pingyu			Yuanyang			Xinyang		
	F_maker_	*P*_marker_	R^2^	F_maker_	*P*_marker_	R^2^	F_maker_	*P*_marker_	R^2^	F_maker_	*P*_marker_	R^2^	F_maker_	*P*_marker_	R^2^
Hs345	25.46	1.41E-18	0.21	21.52	6.24E-16	0.19	13.41	3.41E-10	0.13	26.44	3.11E-19	0.22	37.34	4.08E-26	0.29
Hs4381	50.30	5.14E-20	0.21	33.86	3.25E-14	0.15	40.31	1.53E-16	0.18	46.18	1.32E-18	0.20	75.46	3.73E-28	0.29
Hs485	26.93	9.51E-16	0.18	21.07	1.32E-12	0.14	21.10	1.27E-12	0.15	21.48	7.82E-13	0.15	36.07	1.98E-20	0.23
Hs205	16.43	1.33E-14	0.18	7.90	4.52E-07	0.10	10.37	2.68E-09	0.13	12.46	3.65E-11	0.15	16.29	1.75E-14	0.18
Hs1036	31.71	2.48E-13	0.16	17.49	5.98E-08	0.09	17.87	4.24E-08	0.10	26.00	3.19E-11	0.13	43.84	1.24E-17	0.20
Hs393	16.01	4.43E-12	0.15	13.34	3.73E-10	0.12	12.19	2.61E-09	0.12	16.74	1.32E-12	0.15	29.18	4.76E-21	0.24
Hs672	17.02	8.43E-13	0.15	11.63	6.73E-09	0.11	16.82	1.16E-12	0.16	20.27	4.38E-15	0.18	26.53	2.58E-19	0.22
Hs1956	26.91	1.50E-11	0.14	22.88	4.93E-10	0.12	16.15	2.05E-07	0.09	24.86	8.80E-11	0.13	40.25	2.29E-16	0.19
Hs377	5.73	5.69E-08	0.14	4.46	6.21E-06	0.11	3.92	4.49E-05	0.10	6.26	7.83E-09	0.15	7.09	3.63E-10	0.17
Y2129	26.65	1.99E-11	0.14	16.79	1.17E-07	0.09	15.29	4.57E-07	0.09	24.86	9.25E-11	0.13	30.65	6.69E-13	0.16
Hs4209	11.18	5.03E-10	0.13	7.07	2.56E-06	0.09	9.28	2.55E-08	0.12	12.37	4.41E-11	0.14	23.92	9.21E-21	0.25
Hs4061	25.55	4.14E-11	0.12	17.49	5.59E-08	0.08	21.42	1.60E-09	0.11	23.28	3.05E-10	0.11	30.72	4.66E-13	0.14
Hs1638	20.72	3.30E-09	0.11	14.13	1.29E-06	0.08	18.27	2.97E-08	0.10	19.25	1.23E-08	0.10	24.86	8.66E-11	0.13
Hs618	22.58	5.71E-10	0.11	12.16	7.75E-06	0.06	7.54	6.19E-04	0.04	17.66	4.78E-08	0.09	22.91	4.26E-10	0.11
Hs1514	19.02	1.53E-08	0.10	15.50	3.69E-07	0.08	6.23	2.20E-03	0.04	14.59	8.48E-07	0.08	14.77	7.19E-07	0.08
Hs4089	19.68	7.67E-09	0.09	18.49	2.25E-08	0.09	6.44	1.80E-03	0.03	17.68	4.71E-08	0.09	13.84	1.61E-06	0.07
Hs425	17.07	8.21E-08	0.08	8.38	2.75E-04	0.04	7.90	4.39E-04	0.04	17.02	8.55E-08	0.08	18.86	1.61E-08	0.09
Hs560	4.53	1.96E-04	0.07	4.98	6.48E-05	0.08	4.69	1.33E-04	0.07	6.43	1.91E-06	0.10	7.36	1.97E-07	0.11
Hs635	13.54	2.12E-06	0.07	13.28	2.72E-06	0.07	10.34	4.30E-05	0.05	18.79	1.71E-08	0.09	31.73	1.98E-13	0.15
Hs02	14.67	7.44E-07	0.07	10.16	5.08E-05	0.05	10.31	4.43E-05	0.05	12.41	6.09E-06	0.06	36.20	4.55E-15	0.16
Hs4265	5.17	1.34E-04	0.06	6.58	7.07E-06	0.08	6.70	5.50E-06	0.08	8.54	1.16E-07	0.10	11.90	1.13E-10	0.14
Hs376	11.29	1.76E-05	0.06	11.04	2.22E-05	0.06	12.53	5.52E-06	0.07	16.27	1.72E-07	0.08	21.26	1.89E-09	0.10
Hs586	11.07	2.15E-05	0.06	11.38	1.60E-05	0.06	7.30	7.82E-04	0.04	10.90	2.52E-05	0.06	20.14	5.06E-09	0.10
Hs4082	12.36	6.41E-06	0.06	8.72	2.00E-04	0.04	18.30	2.69E-08	0.09	15.51	3.44E-07	0.08	20.61	3.33E-09	0.10

The markers associated with the PC trait under 5 environments are listed in the table.

For the OC trait, 9 markers were detected in MLM model and 8 markers of Hs345, Hs4381, Hs485, Hs1036, Hs4061, Hs635, Hs376 and Hs586 were confirmed using GLM and MLM methods. Especially, 4 markers of Hs345, Hs4381, Hs485 and Hs1036 had high R^2^ values (≥10%) under 5 environments (Table 3 and Table S3). For the PC trait, 9 markers were found and confirmed in both models, of which 7 markers had high R^2^ values (≥10%) under 5 environments (Table 4 and Table S3). These results suggested that the OC and PC traits are controlled mostly by major genes in sesame.

### Marker effect on the phenotypic variation

To explore the association between the above markers and phenotypic variation and the utility potential in sesame MAS breeding program, we performed the allelic effects of the five markers associated with both traits (Table 5). For each marker, the effects estimated were in accordance with the allele character under the 5 environments. Hs345 had the largest effect on the variation of seed oil and protein content. The Hs345-1∶1 and Hs345-2∶2 of Hs345 showed the different variation effects on OC trait, as the average oil content in the accessions ranged from 52.05%–42.82%. In the genotypes carrying the Hs4381-1∶1 allele, oil and protein contents were 43.89% and 23.25%, respectively, while the samples with the Hs485-2∶2 contained 52.00% oil and 21.00% proteins. Furthermore, the specific allele indicated the negative or positive effect to a large extent on the OC or PC trait. For example, the allele effect of Hs345-2∶2 on OC trait ranged from −8.13 to −12.18, whereas the effect on PC trait ranged from 2.05 to 3.82. Comparison results suggested that the alleles of all 5 markers give the opposite effects on OC and PC traits, respectively. The allele that increased the oil content certainly gave the negative effect on protein content, and vice versa. Therefore, these markers could be used for screening sesame lines with either high oil or protein content.

**Table 5:** Allele effects of 5 markers simutaneously associated with OC and PC traits under 5 environments.

Marker	Allele^a^	2011				2012					
		Pingyu		Yuanyang		Pingyu		Yuanyang		Xinyang	
		OC	PC	OC	PC	OC	PC	OC	PC	OC	PC
Hs345	1∶1	−0.04	−0.17	−0.59	0.25	−1.17	0.44	−1.61	0.57	−0.93	0.39
	2∶2	−10.74	2.30	−8.13	2.12	−8.87	2.05	−12.18	2.96	−11.73	3.82
	1∶2	−5.53	1.38	−4.59	1.67	−5.13	1.29	−5.84	2.08	−6.66	2.58
Hs4061	1∶1	0.00	0.00	0.00	0.00	0.00	0.00	0.00	0.00	0.00	0.00
	2∶2	8.78	−2.41	4.46	−1.95	6.37	−1.84	8.54	−2.33	7.94	−2.96
	1∶2	2.58	−0.98	−0.18	−1.06	1.78	−0.80	2.58	−1.14	1.60	−0.86
Hs1956	1∶1	1.12	−0.36	1.01	−0.49	0.85	−0.32	1.18	−0.39	1.67	−0.81
	2∶2	−5.89	1.21	−4.13	0.79	−4.03	0.63	−5.03	0.95	−5.15	1.34
	1∶2	−0.74	0.15	0.11	−0.28	−0.86	0.26	−0.60	0.49	−0.85	0.02
Hs4381	1∶1	0.00	0.00	0.00	0.00	0.00	0.00	0.00	0.00	0.00	0.00
	2∶2	9.75	−2.45	6.33	−1.73	6.97	−1.82	9.06	−2.17	9.80	−3.41
	1∶2	5.02	−1.05	2.34	−0.49	3.16	−0.90	5.12	−0.75	4.58	−1.51
Hs485	1∶1	−4.80	0.47	−3.23	0.25	−3.87	0.51	−6.88	1.62	−7.19	2.24
	2∶2	2.04	−1.28	0.82	−1.21	1.71	−0.96	−0.02	0.02	0.20	−0.29
	1∶2	−3.99	0.33	−3.85	−0.03	−2.59	−0.12	−4.64	1.38	−5.76	1.74

Note: ^a^ two of the most common alleles are listed.

### Comparative genome analysis

To clarify the distribution and more information of the above associated markers, we performed the comparative genome analysis of the 19 SSR markers associated with the OC trait (Table 6). All genes closest to the markers were explored. Of the 19 markers, 11 are located in gene regions and 8 are in intergenic regions. The flanking genes had various functions, such as ligase (C01.560), transcription factor (C04.26, C13.438) and kinase (C14.22). Moreover, we found that the markers of Hs4082 and Hs345 were just located next to C01.883 (ABC transporter G family member 3 gene) and C04.786 (Stearoyl-ACP Desaturase gene), respectively, which were proved involved in plant lipid biosynthesis. We also screened the upstream and downstream sequences of 500 Kb far from each marker. 17 (out of 19) markers were proved close to plant lipid pathway genes. These result further confirmed our association mapping conclusions.

**Table 6:** Information of candidate genes related to the markers associated with OC traits.

Marker	Related gene^a^	Annotation	Nearby lipid genes^b^	*Arabidopsis thaliana* homologous genes		Ref.
				Loci^c^	Annotation	
**Hs4381**	C01.460	Thioredoxin M3	C01.526	At1G55260	Lipid Transfer Protein type 5	[25]
**Hs02**	C01.560	E3 ubiquitin-protei	C01.548	At3G25110	Acyl-ACP Thioesterase A	[26]
		ligase RHF2A	C01.575	At3G07400	Lipid Acylhydrolase-like	
	C01.561	Unknown	C01.601	At2G30720	Acyl-CoA Thioesterase	[27]
**Hs4082**	C01.882	Cell cycle protein	C01.873	At1G53390	ABC Transporter	[28]
	C01.883	ABC transporter G	C01.883	At2G28070	ABC Transporter	[28]
		family member 3	C01.928	At3G44830	Diacylglycerol Acyltransferase	[29]
**Hs635**	C02.696	RAS-related protein Rab11A	C02.739	At1G47620	Midchain Alkane Hydroxylase	30]
**Hs4209**	C04.25	Transmembrane domain	C04.38	At1G31770	ABC Transporter	[31]
		containing protein	C04.56	At1G71010	Phosphatidylinositol-Phosphate Kinase	[32]
	C04.26	TCP transcription factor 12	C04.81	At1G10900	Phosphatidylinositol-Phosphate Kinase	[32]
			C04.96	At1G77660	Phosphatidylinositol-Phosphate Kinase	[32]
**Hs4061**	C04.681	Inositol trisphosphate 5-phosphatase 1	—			
**Hs345**	C04.785	Unknown	C04.767	At1G05630	Phosphoinositide 5-Phosphatase	—
	C04.786	Stearoyl-ACP desaturase	C04.786	At2G43710	Stearoyl-ACP Desaturase	[33]
**Hs1956**	C04.845	FAR1-related sequence	The same as Hs345			
**Hs393**	C13.438	bZIP transcription	C13.388	At1G10900	Phosphatidylinositol-Phosphate Kinase	[32]
		factor 40	C13.471	At5G10160	Hydroxyacyl-ACP Dehydrase	[34]
			C13.504	At1G17840	ABC Transporter	[35]
			C13.514	At4G36480	Subunit of Serine Palmitoyltransferase	[36]
**Hs205**	C14.21	Thylakoid membrane	C14.49	At4G33550	Lipid Transfer Protein type 3	[37]
		phosphoprotein	C14.66	At1G49430	Long-Chain Acyl-CoA Synthetase	[38]
	C14.22	Serine/threonine-protein	C14.111	At2G46210	Sphingobase-D8 Desaturase	[39]
		kinase WNK4	C14.132	At2G46090	Diacylglycerol Kinase	—
**Hs672**	C14.367	Cyclin-P3.1 F-box	C14.359	At2G45150	CDP-DAG Synthase	[40]
		protein	C14.413	At4G33355	Lipid Transfer Protein type 1	—
	C14.368	Unknown	C14.428	At2G44810	Acylhydrolase (DAD1-like)	—
**Y2129**	C15.825	Unknown	C15.92	At2G29980	Linoleate Desaturase	[41]
**Hs1638**	C15.840	Unknown	The same as Y2129			
**Hs618**	C25.41	IAA-alanine resistance	C25.69	At5G08415	Lipoate Synthase	[42]
		protein 1				
	C25.42	Beta-D-xylosidase				
**Hs376**	C26.474	ADP-ribosylation factor	C26.417	At1G15110	Base-Exchange-type Phosphatidylserine	[43]
					Synthase	
			C26.454	At1G71010	Phosphatidylinositol-Phosphate Kinase	—
			C26.515	At1G31812	Acyl CoA Binding Protein	[44]
**Hs377**	C26.475	Unknown	The same as Hs376			
**Hs485**	sf00001.885	Unknown	sf00001.95	At4G04930	Dihydrosphingosine Delta-4 Desaturase	[45]
	sf00001.886	Unknown				
**Hs586**	sf00001.754	ADP-ribosylation factor	—			
**Hs1036**	sf00044.12	ABC transporter I	sf00044.12	At3G20320	Acid-Binding Protein	[46]
		family member 15	sf00044.41	At2G25170	Chromatin remodeling factor	[47]
			sf00044.61	At4G19860	Acyl acceptor Acyltransferase	—
			sf00044.90	At1G13210	Translocase	—
			sf00044.113	At1G74320	Choline Kinase	[48]

Note: ^a^Related genes are the genes containing or close to the screened markers.

b Nearby lipid genes refer to the genes located in the upstream and downstream of 500 Kb far from the marker.

c Only one of the homologous genes is listed in the table.

— refers to no known lipid genes in the location.

## Discussion

To clarify the genetic mechanisms of fatty acid and protein synthesis in sesame seeds, we herein performed the association mapping analysis of the OC and PC traits among 369 sesame accessions using 112 genic-SSR markers. These accessions were collected from 19 provinces of China and 15 other countries, and represented the genetic diversity of sesame germplasm for association mapping study. These accessions included many released Chinese and foreign cultivars. Compared with the traditional linkage analysis (QTL mapping), the association analysis based on linkage disequilibrium (LD) has been applied for the quantitative trait loci (QTLs) detection and location in many crops. Meanwhile, GLM and MLM models are applied individually for evaluating the marker association. In this article, 19 SSR markers associated with the OC trait were detected under each 5 environments in GLM model, while 24 markers were determined and associated with the protein content.

### Sesame genetic diversity and the population structure

Sesame is a diploid and self-pollinated oil crop with the karyotype of 2n = 2x = 26. As all cultivars are originated from the sole cultivated species, *Sesamum indicum* L, the narrow genetic diversity presents in regional sesame resources to a large extent [24], [49]–[51]. In addition, many reports reflect that there is no clear association between genotype and geographical origin, as many sesame accessions from the different geographic locations are clustered into the same group in the dendrogram [51]–[53]. Apart from the natural history of predomesticated ancestors, the diversity pattern of domestic species could be influenced by the breeding practice, germplasm collection and human activity [53]–[55]. In this article, in order to guarantee the broad genetic variation, we selected the 369 worldwide accessions for seed nutrition genetic analysis according to the geographical origin, molecular clustering and the morphologic diversity (Table S1). The heterozygosity ranged from 0.27% (Hs373)-23.12% (Hs4325). The result proved that the natural population could be used as the core germplasm for association mapping (Table S2). Population structure analysis showed that many sesame accessions collected from the same geographic locations were not grouped together, which further proved the unclear association between genotype and geographical origin in sesame germplasm resource [51]–[53].

During performing the association mapping in a population, LD patterns between the functional loci and markers should be analyzed at first [56]. We analyzed the *P*-values of linkage disequilibrium between the polymorphisms of the 112 SSR marker loci using Fisher's exact test (Figure 1). As 363 (5.84%) pairwise comparisons had the high LDs (*D*′>0.5), the linkage disequilibrium existed in 369 sesame accessions. Accordingly, we believed that the natural population is suitable for association analysis of the oil and protein contents.

### Oil and protein content variation and associated SSR markers

Among large-scale sesame germplasm resources, the oil and protein contents varied significantly. Yermanos et al. [57] evaluated 721 sesame samples collected from more than 19 countries, and found that the oil content varied from 40.4–59.8% with the mean of 53.1%. The protein content ranged from 19–31% with an average of about 25% [58]. In our population, the oil content varied from 27.89%–58.73% with an average of 51.34%, while the protein content varied from 16.72%–27.79% with an average of 21.19% (Table 1). The data reflected the great variation of sesame seed compositions in germplasm [57], [59], [60].

Comparison analysis proved that there is a strong negative correlation of the oil content with protein content. Interestingly, the stringency relationship was also exhibited in the association analysis. As shown in the GLM analysis in Table 3 and 5, all the 19 markers associated with OC were detected for PC trait; and the alleles exhibited the opposite effects on OC and PC at the same time. These phenomena also present in other oil crops, such as oilseed rape, cotton, soybean and peanut [1], [61]–[63]. Zhao et al. [62] found 6 QTLs with pleiotropic effects on both oil content and protein content in oilseed rape. In the cotton backcross inbred population, of 17 QTLs for oil content and 20 QTLs for protein content, 8 QTLs co-localized in the same chromosome regions and controlled oil and protein contents with opposite additive effects [63]. Hwang et al. (2014) detected 25 SNPs associated with seed oil in 13 different genomic regions through GWAS (genome wide association study), and 7 SNPs were significantly associated with both protein and oil traits. For the six of seven marker loci, a negative relationship existed between the protein effect versus that on oil [64]. Meanwhile, QTLs or markers associated with increased protein and oil contents were also found. For example, Zhao et al. (2006) found that 2 QTLs that controlled oil content were independent from protein content by conditional QTL mapping [65]; Hwang et al. (2014) found a SNP at the 4.92 Mb position on Chr 9 was associated with increased protein and oil contents [64].

In many crops, the seed oil content seems to be controlled mostly by major genes [66]–[69]. In this study, we detected 19 markers associated with OC trait in sesame using GLM method, and the R^2^ values ranged from 4.0–29.0%. Chen et al. (2010) screened 27 QTLs related to oil content in oilseed rape and the individual explanation was high with the range of 4.20–30.20% [66]. In *Arabidopsis thaliana*, a single QTL or marker could give an explanation of 17–19% for the seed oil content [68]. In soybean, the explanation reached to 14.3–45.6% [69]. In this report, 112 polymorphic SSR markers were used for association mapping. Compared with other common molecular markers, such as SRAP and AFLP, SSR marker is more suitable for sesame diversity analysis due to the narrow genetic basis [24], [49]–[51]. The marker distribution and density were analyzed using the sesame genome assembly data. The contigs/scaffolds carrying the 112 markers approximately covered 180 Mb of the sesame genome and occupied ∼67% of the assembly size (270 Mb) and 50% of the estimated genome size (∼360 Mb, in which 90 Mb was believed to be repeat sequences) (Table S2) [70], [71]. Therefore, the association mapping using 112 markers is meaningful and reliable, even though some QTLs might be missed. To detect more QTLs, new SSR or SNP markers could be applied in further research.

### Candidate genes and oil components

To clarify the marker location and more genome information for OC and PC traits, we screened the genes that were close to the associated markers using the sesame genome assembly data. As a result, 36 candidate genes related to lipid pathway were identified (Table 6) [25]–[48]. We found that most candidate genes were involved in three pathways: (1) fatty acid and TAG (triacylglycerol) synthesis and elongation, e.g., C01.526, C01.548 and C01.928; (2) TAG degradation, e.g., C01.601; and (3) fatty acid dehydrogenation, e.g., C04.786 (Stearoyl-ACP desaturase, determining the ratio of saturated and unsaturated fatty acids) (Table S4). Therefore, the seed oil content in the sesame accessions could be regulated by three factors, i.e., oil synthesis ability, oil degradation ability and oil component ratio (e.g., 18∶1 and 18∶2 fatty acids). In various accessions, any alleles of the genes involved in fatty acid and TAG synthesis, TAG degradation or dehydrogenase genes could give effects on oil content. To confirm this hypothesis, further studies of seed oil synthesis should be performed in the future.

## Conclusion

We systematically explored the association mapping of seed oil and protein content traits in 369 worldwide sesame accessions using the 112 SSR markers. A significant negative correlationof the oil content with the protein content existed in the population. 19 SSR markers were associated with the oil content trait with high phenotypic variation explanation, and 24 SSR markers were associated with the protein content trait using GLM method. This association results would provide an efficient platform for seed development research and MAS breeding in sesame.

## Methods

### Plant materials

A population of 369 core sesame germplasm resources was chosen according to the genetic diversity analysis results and phenotypic variation [51]. These core genotypes comprised 318 lines from 19 provinces of China and 51 worldwide lines from the 15 countries, which were reserved at the Sesame Germplasm Bank of Henan Sesame Research Center (HSRC), HAAS (Henan Academy of Agricultural Sciences) (Table S1). All accessions were grown with three replications at three different experimental stations of Yuanyang (113.96°E, 35.05°N), Pingyu (114.62°E, 32.97°N), and Xinyang (114.08°E, 32.13°N) in 2011 and 2012. Five or six young leaves of individual accessions were collected and reserved at −70°C for DNA extraction.

### Oil and protein content analysis

After harvested, ∼20 g of seeds were collected from five plants per line, and the seed OC and PC were measured on infrared determination equipment (Perten DA7250, Sweden) according to the manufactures' instructions. The standard curve for measuring the sesame oil and protein contents had been established according to the chemical analysis results of 300 sesame accessions (Unpublished data, HSRC). Three replications of each samples were assayed for phenotypic analysis. Mean value, broad-sense heritability and correlation coefficient of the phenotypic data were analyzed using the statistical analysis system software (SAS Institute Inc. 2002) [72].

### SSR genotyping

The 112 polymorphic SSR pairs were selected from our SSR marker bank [24], [51], [73] (Table S2). DNA extraction, PCR amplification, electrophoresis and SSR genotyping analysis were performed according to the methods described by Zhang et al. [24]. The total number of polymorphic alleles at each SSR locus was calculated according to the results of all 369 lines. The polymorphic SSR alleles presented only within 4 (1%) or fewer accessions were recorded as rare alleles.

### Linkage disequilibrium (LD)

As the population structure could result in the spurious associations between phenotypes and marker loci, we analyzed the extent and structure of LD within the population before selecting the appropriate association mapping strategy. To assay whether the 112 polymorphic SSR markers were segregated independently or not, LD analysis was conducted according to the dedicated procedure of the TASSEL software [49]. Both *D*′ and r^2^ were used for quantifying LD values [74], [75]. Significance (*P* values) of *D*′ for each SSR pairs was determined with 100,000 permutations.

### Population structure and relatedness analysis

The population structure was determined using STRUCTURE 2.2 [76]. The mixture model and the independent allele frequency model were used to analyze the population dataset. Five runs of STRUCTURE were carried out for each number of populations (*K*) (from 1–10), and each run started with 10,000 burn-ins, followed by 100,000 iterations. While performing the STRUCTURE, we assumed that the inferred population accord with Hardy Weinberg equilibrium (HWE) and the loci are unlinked. To correct the relatedness of individuals in further analyses, the relatedness between individuals (relative kinship) was evaluated using SPAGeDi 1.2 software [77]. The matrix with the relative kinship coefficients (*K* matrix) was applied for association analysis using the Mixed Linear Model(MLM, Q+K)method.

### Association mapping and marker distribution in sesame genome

Associations between the SSR markers and the oil and protein content traits were investigated using both methods of the general linear model (GLM, Q) and the mixed linear model (MLM, Q+*K*) in TASSEL 2.1 described by Bradbury et al. [49]. The mean value of the markers at *P*<0.005 was used for determining the significance of marker-trait associations.

To determine distributions of the associated markers in sesame genome, we carried out the alignment of SSR markers and transcripts with the updated sesame genome data [70], [71]. In the present genome assembly, the number of N50 scaffold was 14, and the number of N90 scaffold was 64. 29,798 gene models were identified. Among related scaffolds or contigs, the sesame lipid synthesis related genes were identified according to the homologous comparison using the genes of *A. thaliana* from the Acyl Lipids pathway database [78] as queries.

## Supporting Information

Table S1 Origins of 369 sesame accessions.
(DOCX)

Table S2 Diversity statistics of 112 SSR markers in 369 sesame accessions and locations in the sesame draft genome
(DOCX)

Table S3 Association mapping of the OC and PC traits using MLM method.
(DOCX)

Table S4 Functions and involved pathways of the candidate genes.
(DOCX)
