# Supplementary material for: Association Mapping of Seed Oil and Protein Content in Sesamum indicum L. Using SSR Markers
Source: PLoS One. 2014 Aug 25;9(8):e105757. doi: 10.1371/journal.pone.0105757 (PMC4143287; doi:10.1371/journal.pone.0105757)
Supplement: Table S1 — Origins of 369 sesame accessions. (DOCX) [file pone.0105757.s001.docx]

**Table S1 Origins of 369 sesame accessions**

| **Origin** | **Number** | **Origin** | **Number** |
| --- | --- | --- | --- |
| Henan, China | 70 | Xinjiang, China | 2 |
| Hubei, China | 58 | Zhejiang, China | 1 |
| Anhui, China | 21 | Mozambique | 3 |
| Shanxi, China | 17 | Japan | 4 |
| Liaoning, China | 22 | Burma | 4 |
| Hebei, China | 25 | Venezuela | 2 |
| Jiangxi, China | 35 | Russia | 1 |
| Shandong, China | 11 | Guinea | 3 |
| Jilin, China | 13 | Turkey | 1 |
| Shaanxi, China | 3 | Vietnam | 2 |
| Guangdong, China | 20 | Thailand | 2 |
| Jiangsu, China | 3 | Greece | 3 |
| Guangxi, China | 7 | UAE | 4 |
| Guizhou, China | 6 | Korea | 3 |
| Tianjin, China | 1 | India | 3 |
| Sichuan, China | 1 | USA | 10 |
| Yunnan, China | 2 | Mexico | 6 |
