# Supplementary material for: Association Mapping of Seed Oil and Protein Content in Sesamum indicum L. Using SSR Markers
Source: PLoS One. 2014 Aug 25;9(8):e105757. doi: 10.1371/journal.pone.0105757 (PMC4143287; doi:10.1371/journal.pone.0105757)
Supplement: Table S2 — Diversity statistics of 112 SSR markers in 369 sesame accessions and locations in the sesame draft genome (DOCX) [file pone.0105757.s002.docx]

**Table S2 Diversity statistics of 112 SSR markers in 369 sesame accessions and locations in the sesame draft genome**

| **Marker**  **name** | **Forward primer (5’-3’)** | **Reverse primer (5’-3’)** | **Allele**  **No.** | **Frequency**  **of major allele** | **Heterozygosity** | **PIC** | **Gene**  **diversity** | **Location ^a^** | **Contig**  **length (bp)** |
| --- | --- | --- | --- | --- | --- | --- | --- | --- | --- |
| Hs1737 | TCCTCCCCGCTATCTTATTG | CAGTCGACGCAGATCTTGTT | 2 | 0.7515 | 0.0798 | 0.3037 | 0.3735 | C08:8340187 | 9969922 |
| Hs1885 | GTCGGCAACGCAGTTAAGTA | TATATGTAATCTGCCCCGCA | 2 | 0.5821 | 0.0836 | 0.3682 | 0.4865 | C08:5920672 | 9969922 |
| Hs1987 | AGTTGCAATGGCTGGAGATT | CACAGACGACAACAAAACCC | 2 | 0.6353 | 0.1155 | 0.3560 | 0.4634 | C09:12988612 | 15067831 |
| Hs1526 | AGCTATGGGTCCACCAAAAT | GGTTTCTTGCTGCTGCTGTA | 2 | 0.6815 | 0.0714 | 0.3399 | 0.4341 | C04:14515590 | 1785203 |
| Hs1444 | GTTCGAGCCCTTCAAGATTC | AGCCCACGAAACTCTTCCTA | 2 | 0.6168 | 0.1138 | 0.3610 | 0.4727 | C22:1790769 | 8599974 |
| Hs1803 | TTAGACTGGGAAGTGGGGAC | GGTGGTATTCGAGCTGGTG | 2 | 0.5595 | 0.1860 | 0.3714 | 0.4929 | C03:5077516 | 12376013 |
| Hs1656 | CAAGCTGGGATATTCCCCTA | CGTAGCTGTAACGGAGTTGC | 2 | 0.5495 | 0.0836 | 0.3725 | 0.4951 | C03:3067820 | 12376013 |
| Hs1956 | CACAGTTACCATGGGCAAAG | ACACCCATATTTCCAGGCAT | 2 | 0.8619 | 0.0541 | 0.2098 | 0.2381 | C04:5955178 | 1785203 |
| Hs1514 | CCATGAGTGTTGGCTTTCTC | CTGCTGTAAATGACGGGATG | 2 | 0.5913 | 0.1347 | 0.3665 | 0.4833 | C19:2141324 | 3786393 |
| Hs1800 | GACTGCACGGACTTGAAGAA | CATGGACCATCCATCACATT | 2 | 0.9036 | 0.0326 | 0.1591 | 0.1743 | C10:3005830 | 4071413 |
| Hs1832 | TAACGGGTAGGTTTCGATCC | AGAAAGGCAAAGCAGAGGAG | 2 | 0.6269 | 0.1146 | 0.3584 | 0.4678 | Scaffold00019:  1787073 | 2909052 |
| Hs1814 | AACCAACACAAAGCTTGCAC | GTGTCCAAAACAGCAAATGG | 4 | 0.8201 | 0.0183 | 0.2613 | 0.2992 | C22:1077940 | 8599974 |
| Hs1917 | GTTGAAAGAGCAAACCGACC | CCACGGAGGACTGCTTTTAT | 2 | 0.9659 | 0.0148 | 0.0637 | 0.0659 | C18:5285160 | 6977756 |
| Hs1977 | ACAGTATTTGGGACTTGCCC | GCCGTTTCCTTGTCTGATTT | 2 | 0.6743 | 0.1131 | 0.3428 | 0.4392 | C02:5871704 | 6665137 |
| Y2129 | GGGGCACAGAGTGGATGTAG | GGACCATGTAATCCCAGCAC | 2 | 0.8034 | 0.0526 | 0.2660 | 0.3159 | C15:5571105 | 5953873 |
| Y1999 | CATTAGGCCTTGTCCATGCT | CAATAACCCGTGAGGTGGAG | 2 | 0.7774 | 0.0564 | 0.2862 | 0.3461 | C05:7740956 | 8464422 |
| Y1972 | CACGGAAGCAGCTCATCAT | CCTGCCGACATGACTACAAC | 3 | 0.6404 | 0.0946 | 0.3586 | 0.4628 | C03:3323206 | 12376013 |
| Y1985 | TAGCTCTCGCCGTTCTGTTT | CTCCTCCTCGAACCTTCCTT | 3 | 0.7461 | 0.0805 | 0.3104 | 0.3804 | C03:5421828 | 12376013 |
| Hs1623 | CTCTACTTGAATCCGAGCGA | CAAGGTTTGATGAGCTCGAA | 3 | 0.6994 | 0.0467 | 0.4093 | 0.4599 | C14:297023 | 8468986 |
| Hs1703 | CCATTATTAGCAGCAACCCA | GGGGAGAAAGGGAGAGAAAC | 2 | 0.6526 | 0.0841 | 0.3506 | 0.4534 | C16:1952819 | 7395225 |
| Hs1909 | GTAACAGGGGATTTCATGGG | CTGGAATTCTGAAAGACCCG | 2 | 0.5612 | 0.0765 | 0.3712 | 0.4925 | C04:1004690 | 1785203 |
| Hs1972 | TTCGGTGGCATTAGCTGTAG | TTGTGGGCATAGGTGGTAGA | 3 | 0.4955 | 0.0964 | 0.4449 | 0.5476 | C03:4545036 | 12376013 |
| Hs1961 | GCAAGAAGTCAGCTGATGGA | TTCACATTCAAGTCAAGCCC | 2 | 0.6115 | 0.0764 | 0.3623 | 0.4752 | Scaffold00044:  1506331 | 1683296 |
| Hs1577 | TGGACGAGTTCAACCCAATA | GATATTCAAGGCCCATGACC | 2 | 0.8555 | 0.0472 | 0.2167 | 0.2473 | C12:358250 | 6723804 |
| Hs1775 | CAACCCATGATACAGCAACC | TTCCAGATGCACCTCCATTA | 3 | 0.9249 | 0.0120 | 0.1364 | 0.1415 | C01:4140354 | 8650717 |
| Hs1792 | ACCCTGGCGTAAAAGACTTG | TCCAGAGAGAGAAACCCAAGA | 3 | 0.8063 | 0.0313 | 0.2959 | 0.3265 | C11:1318715 | 9215640 |
| Hs1576 | GGTGAATGGTTTTGTGTGGA | TAAAGCCATGACGAAGCAAG | 4 | 0.4741 | 0.1189 | 0.4581 | 0.5570 | C09:8897068 | 15067831 |
| Hs1545 | GTCATCATCACAGCCCCAT | GGAGTTCTGTGAGGGTGGTT | 3 | 0.5016 | 0.1188 | 0.5005 | 0.5860 | Scaffold00106:  773782 | 962480 |
| Y1994 | CTGGGGAAGGAAGTGGTGTA | TTGCAGAAGCCTTAACAGCA | 4 | 0.3208 | 0.1778 | 0.6692 | 0.7221 | C14:5646981 | 8468986 |
| Hs1180 | TTTCGTTGGGAGAAGAAGAG | TGTCTTTCACCAAACGCCTA | 3 | 0.4164 | 0.1293 | 0.5485 | 0.6276 | C01:685126 | 8650717 |
| Hs1373 | GGTCGGATCTTCAGACCTGT | ACGACGCTAAGTCCGAATCT | 2 | 0.9856 | 0.0000 | 0.0280 | 0.0284 | C092276564 | 15067831 |
| Hs1163 | CATCAGGCCAATCCCTATCT | ATGCTATCCCCACCACTCTC | 3 | 0.9005 | 0.0627 | 0.1645 | 0.1797 | Scaffold00001:  2766144 | 7967487 |
| Hs1178 | TCTTGTTCATTTGGCAGCTC | CTCATGTGCCTTCTGCTGAT | 2 | 0.8811 | 0.0732 | 0.1876 | 0.2095 | C02:3296799 | 6665137 |
| Hs1082 | GATTGCCTGGACAAGAGGAG | ATGAGAAGCTTGGGTCCTGT | 2 | 0.7632 | 0.0695 | 0.2961 | 0.3614 | C04:812608 | 1785203 |
| Hs1638 | TAGGAAGAGGCATGTTCACG | CCATCTCCACATCTTGCATC | 2 | 0.8427 | 0.0831 | 0.2299 | 0.2651 | C15:5686621 | 5953873 |
| Hs1420 | TTGTTCACAGGAAGCGGTAG | ACGCTGTACAAATCCGAAAG | 2 | 0.9449 | 0.0290 | 0.0987 | 0.1041 | C09:9614750 | 15067831 |
| Hs1412 | GGCATTTGCCTCCATTCTAT | GTAAGTGAAGGAGAAGGGGG | 2 | 0.7791 | 0.0478 | 0.2850 | 0.3442 | Scaffold00010:  297864 | 4182001 |
| Hs1564 | GGTTTGGGAGAGCAGTCTTC | CTATCAGGCGCAAGATACGA | 2 | 0.7530 | 0.0783 | 0.3028 | 0.3720 | C15:1636617 | 5953873 |
| Hs1010 | CATAAAGGGCTACGGGGATA | GGCTACAAAGAGGCATCTCC | 2 | 0.6109 | 0.0973 | 0.3624 | 0.4754 | C15:1977504 | 5953873 |
| Hs1036 | GCCTGCTTAGCTGCCTTTAG | CAGACGGAGATGCAGATTGT | 2 | 0.9423 | 0.0385 | 0.1028 | 0.1087 | Scaffold00044:  118246 | 1683296 |
| Hs1385 | GAGAAAGTTCAGGTGTGCGA | TTGTCCAGAAGCCCTTTCTT | 2 | 0.6096 | 0.0673 | 0.3627 | 0.4760 | C04:1081603 | 1785203 |
| Hs1391 | TTATAGGTCTCCCCGTTTGC | GTACTCCGGATTCTTCTCGC | 3 | 0.7939 | 0.0115 | 0.2826 | 0.3311 | Scaffold00145:  265369 | 463734 |
| Hs319 | GACCGAGGAAAGGAAATTGA | AAACACCAGCTGCAGACAAC | 2 | 0.8062 | 0.1057 | 0.2636 | 0.3124 | C04:1789712 | 1785203 |
| Hs311 | TTGGGTTGTGTCAGAAATGG | CAGTTGTCCTGGGATGAAGA | 2 | 0.9187 | 0.0488 | 0.1382 | 0.1494 | Scaffold00010:  3809944 | 4182001 |
| Hs318 | GAGGCACACTCAGCTCTCTG | TAGATTTGTCACTGGCCCAA | 2 | 0.9702 | 0.0163 | 0.0562 | 0.0578 | Scaffold00200:  38150 | 59536 |
| Hs330 | TCCCTTCCACTTCTCCTCAT | GTGTCCTCCGTTTCATCCTT | 2 | 0.6233 | 0.1463 | 0.3593 | 0.4696 | C12:824629 | 6723804 |
| Hs334 | GAGGAAAGATCAGCGGAAAC | AAACAGCAGCACTATGTCGG | 2 | 0.5854 | 0.1734 | 0.3676 | 0.4854 | C04:754593 | 1785203 |
| Hs340 | ATCTTGTTCTCGATCCGCTT | CCCACCTCTCCTTCTCATCT | 2 | 0.8220 | 0.0788 | 0.2498 | 0.2926 | C18:3808630 | 6977756 |
| Hs345 | TCTCGCTTCTTTGAGAGCTA | GCTACGCCAAACTCATTTCA | 3 | 0.9123 | 0.0712 | 0.1517 | 0.1617 | C04:5378488 | 1785203 |
| Hs350 | CTCAGACCACACCGTTTCAC | CGCCTTCCTCAGACTCGTAT | 3 | 0.9125 | 0.0694 | 0.1555 | 0.1629 | C18:4281909 | 6977756 |
| Hs352 | TTCCGCTGCTTGTATGATTC | TGGTGGAAAAAGAAGGGAAC | 2 | 0.8860 | 0.0797 | 0.1816 | 0.2020 | C13:3454192 | 5707146 |
| Hs361 | TCCCCTTTCACTATTTTCCG | AGAAAGAGACAGCAGCAGCA | 2 | 0.9187 | 0.1084 | 0.1382 | 0.1494 | C13:2678347 | 5707146 |
| Hs364 | AGATTACACGACAACGCTGC | TTCTTGGTTGGTCTGCCATA | 3 | 0.5383 | 0.1612 | 0.4529 | 0.5481 | C13:1690307 | 5707146 |
| Hs375 | CGGAGTTGGAGGAAGAGAAG | GGGATGATCCACCATTGATA | 2 | 0.9255 | 0.0623 | 0.1284 | 0.1379 | C01:8142979 | 8650717 |
| Hs376 | AATGTGGCCAAGTTGAGGTT | AAACAGCAAACTGGTGCTTG | 2 | 0.9780 | 0.0110 | 0.0422 | 0.0431 | C26:3374735 | 7452264 |
| Hs378 | CTCAATCCAACACCCACAAC | TGTAGGGGTGGGAGAAAAAG | 4 | 0.7100 | 0.1192 | 0.3421 | 0.4193 | C03:5243750 | 12376013 |
| Hs393 | GACTGAGTTTGCAGCGAAAG | GTGCTGCACACAGACACAAG | 3 | 0.8577 | 0.1003 | 0.2229 | 0.2476 | C13:2721888 | 5707146 |
| Hs395 | GGAAGCTCTGGACCAAAGAG | GGCTGTGGAAGAGAAGGAAG | 2 | 0.7818 | 0.1274 | 0.2829 | 0.3411 | C18:4247860 | 6977756 |
| Hs398 | ACATCACATCACTGCCAACC | GCGCAGAAATATCCTGTGAA | 2 | 0.8089 | 0.0949 | 0.2613 | 0.3091 | C01:8044956 | 8650717 |
| Hs418 | GCTGCCATTTCTTCTTGTGA | GAAAACCACCCAACTTGAGA | 2 | 0.7458 | 0.1039 | 0.3073 | 0.3792 | C19:2416751 | 3786393 |
| Hs425 | CGGCCCCACTCTAACAAAT | TTGCCCAATTAAGTCAACCC | 2 | 0.8320 | 0.0650 | 0.2405 | 0.2796 | C08:9734347 | 9969922 |
| Hs464 | CTTCTCCAACTTCTCCCTCG | CTCCCCAAGAAATCTGGAAA | 2 | 0.7802 | 0.1264 | 0.2841 | 0.3430 | Scaffold00229:  43958 | 46251 |
| Hs467 | TAGCTAGCCATGCCATCAAC | CATGAAATCACCCATCATCC | 2 | 0.9713 | 0.0137 | 0.0542 | 0.0557 | C12:3787743 | 6723804 |
| Hs474 | AGGAACGGGTCATCAGATTC | TCGCATTCGTTATTCGGTAG | 2 | 0.5313 | 0.1984 | 0.3740 | 0.4980 | C04:14112251 | 17185203 |
| Hs485 | AGAGCTGATGGAAAGGAGGA | TCTCCATCTGTTCGTCTTCG | 3 | 0.9011 | 0.0623 | 0.1637 | 0.1788 | Scaffold00001:  6921992 | 7967487 |
| Hs486 | TAGGACTTGACCTCCTTGGG | CACACAAAATCGCCACTCTC | 2 | 0.6785 | 0.1199 | 0.3411 | 0.4363 | Scaffold00126:  76100 | 431126 |
| Hs487 | CACACAAAATCGCCACTCTC | TAGGACTTGACCTCCTTGGG | 2 | 0.6826 | 0.1226 | 0.3394 | 0.4333 | Scaffold00126:  76236 | 431126 |
| Hs505 | TTACTCCACCCTCACAAAACC | AATAGACATGGGCTCCAACC | 3 | 0.6322 | 0.1144 | 0.3605 | 0.4671 | Scaffold00044:  1545883 | 1683296 |
| Hs517 | TTCTGATGAGCCACCCATTA | AGCAGCCAGAAAGTGGTTTT | 3 | 0.5562 | 0.1370 | 0.3796 | 0.4985 | C09:5066725 | 15067831 |
| Hs560 | AAACAACAGCCCAGATTTCC | TGTCAGGCAACTCATCATCA | 4 | 0.7645 | 0.1108 | 0.3640 | 0.3919 | C11:1763565 | 9215640 |
| Hs561 | GGTGAGAGGAAGTGGGGTAA | CAACAACACCATCAATTGGG | 3 | 0.5217 | 0.1821 | 0.4138 | 0.5247 | C16:1247023 | 7395225 |
| Hs563 | ACATGCACGGGTCAAGTAAA | ATAAAAACCCGTCAGAAGCG | 2 | 0.8306 | 0.0929 | 0.2418 | 0.2814 | C11:4420106 | 9215640 |
| Hs568 | ACGGATCGGGATTTCAATAG | GGCTCTCCACCATTTTGACT | 2 | 0.8641 | 0.0870 | 0.2072 | 0.2348 | C14:5964068 | 8468986 |
| Hs577 | CTCTCTCTCTCACGCATCCA | CGAGTTTAAAGGGGAGACCA | 3 | 0.5027 | 0.1401 | 0.4857 | 0.5754 | C04:14102054 | 1785203 |
| Hs578 | GAAATTTTATGTGGGGGTGC | TGGGTGCGTGCAGAATTA | 4 | 0.5136 | 0.1924 | 0.3848 | 0.5061 | C04:14100732 | 1785203 |
| Hs586 | CACTGGGTCTCCTCCACTCT | ACTCCACCTTCAAATCCCAG | 2 | 0.9553 | 0.0352 | 0.0818 | 0.0854 | Scaffold00001:  5925309 | 7967487 |
| Hs618 | CTCCCACTCACATGATGACC | CAAGCATTTTCTCCACCAGA | 2 | 0.9470 | 0.0516 | 0.0953 | 0.1004 | C25:277159 | 8051950 |
| Hs612 | GGCCTTGTCAGTTTCCTCAT | CACCTCCAGCAACAAGAAGA | 2 | 0.5938 | 0.1549 | 0.3661 | 0.4824 | C08:8410925 | 9969922 |
| Hs616 | ACCCAGTCAACACCTTCTCC | CGCCAAGTACTGAAAAACGA | 2 | 0.6335 | 0.1335 | 0.3565 | 0.4643 | Scaffold00134:  202296 | 302586 |
| Hs628 | CAAGCAACCCTTATCCCAGT | TATGGTGGTATTCACCACGG | 2 | 0.8139 | 0.0027 | 0.2571 | 0.3030 | C14:3583924 | 8468986 |
| Hs635 | ATGAAGACATTCAACGCTGC | CTCCACTACTCGCCATTTCA | 2 | 0.7038 | 0.1359 | 0.3300 | 0.4169 | C02:5871791 | 6665137 |
| Hs665 | CTCAAACACTTTTCCCCACA | TCACACACCAGTGCTTCAGA | 2 | 0.9414 | 0.0300 | 0.1042 | 0.1103 | C05:5524828 | 8464422 |
| Hs674 | GCAACCTCGTCGAATCATAA | TATGTCACAATCAGCGGGAT | 2 | 0.7615 | 0.1084 | 0.2973 | 0.3632 | Scaffold00001:  711763 | 7967487 |
| Hs672 | CACACACTCACCAACCCTCT | CAGGGATGCAAGAACTGAAA | 3 | 0.8875 | 0.0732 | 0.1898 | 0.2036 | C14:1813367 | 8468986 |
| Hs21 | CGGAATTCCTGAAAGAAGGA | CAGTGAATTTCTCAACCCGA | 4 | 0.5870 | 0.1821 | 0.4975 | 0.5638 | C03:5506334 | 12376013 |
| Hs207 | TCCTCACCATCATTGCTCAT | CTAAACTCAAGAAAGCGGGG | 2 | 0.5272 | 0.1335 | 0.3743 | 0.4985 | C03:4545063 | 12376013 |
| Hs53 | GAAGCTTGAAGAGAGGAGGG | ATGGAACTTCTCCGATCACC | 2 | 0.6359 | 0.1793 | 0.3559 | 0.4631 | C04:3459706 | 1785203 |
| Hs02 | CCATTAAATTCTTGCTCCCC | CTGGTCGTATGCAGCATCTT | 2 | 0.8767 | 0.0786 | 0.1928 | 0.2162 | C01:4431508 | 8650717 |
| Hs205 | GATGTGATGGTGGTGAGAGC | GCTATGCGTTGAATGAAGAC | 3 | 0.8795 | 0.0740 | 0.2080 | 0.2193 | C14:121510 | 8468986 |
| Hs226 | AGGGGAGAATTCAGGTGTTG | GGGCACTGGAGATTTCTGTT | 2 | 0.6951 | 0.1653 | 0.3340 | 0.4239 | C04:14263376 | 7185203 |
| Hs233 | CGTCCCGTGTTGTCTCTATG | GCGGAGAATATGCCGTTATT | 3 | 0.5503 | 0.1440 | 0.3744 | 0.4962 | Scaffold03382:  254 | 2113 |
| Hs235 | TTGCAGTAAAGCAATGGAGG | ATGCAGCATCAACTCCTCAG | 2 | 0.9024 | 0.0759 | 0.1606 | 0.1761 | C03:2994574 | 12376013 |
| Hs250 | ACTTCGGTCCCTCCCTCTT | GGAGTAGAATTTCCGGGTCA | 3 | 0.6599 | 0.1247 | 0.3693 | 0.4602 | C05:5832911 | 8464422 |
| Hs259 | AAAGCCTCCCATACGATCAC | ACCGACGGAAACAACTAAGC | 2 | 0.5163 | 0.1978 | 0.3747 | 0.4995 | C04:14112512 | 1785203 |
| Hs270 | TTCGTGAAAAGGAGTTGTGC | CAGAGGTCACCATTGACGAG | 3 | 0.5761 | 0.1712 | 0.4064 | 0.5110 | Scaffold00173:  51964 | 129636 |
| Hs4089 | GACGCTGTTCCAAAACACAC | CGCACGATTCACTCCTTTTA | 2 | 0.6223 | 0.1250 | 0.3596 | 0.4701 | C19:2036572 | 3786393 |
| Hs4144 | AAAAATACCCTCAAACCCCC | GTCCTTCCTGTCTGAGAGCC | 3 | 0.4945 | 0.1260 | 0.4190 | 0.5298 | C24:4036209 | 9156729 |
| Hs377 | GGATATATGTCCATCGCCCT | CAGCAAGACCAATGCCTCTA | 5 | 0.7451 | 0.1421 | 0.3635 | 0.4063 | C26:3375580 | 7452264 |
| Hs216 | TGAGAGAGGTTAATTGGGGG | TGGCTCCCATGTATTTACCA | 3 | 0.4608 | 0.2213 | 0.5388 | 0.6181 | C16:6120148 | 7395225 |
| Hs4140 | GAACTTCTGCTGAAATTGGG | TGATCTTCACTTTCCCCAGC | 4 | 0.4778 | 0.2250 | 0.5848 | 0.6474 | Scaffold00001:  620506 | 7967487 |
| Hs4061 | TTTGAGCATTCTGTCCTTGC | GACCTGAACTTCATCGGGTT | 2 | 0.9566 | 0.0379 | 0.0795 | 0.0830 | C04:4516824 | 1785203 |
| Hs4387 | ATCCTTCCTTCCACCTCCTT | TCAGCTGAAGGTAATGTGCC | 2 | 0.9458 | 0.1030 | 0.0973 | 0.1025 | C26:4033469 | 7452264 |
| Hs4381 | TGCCTGATGTGTTTGTGAGA | CTCAGGGTCGAATTGTGATG | 2 | 0.9201 | 0.0461 | 0.1363 | 0.1471 | C01:3824440 | 8650717 |
| Hs4265 | CACATCTGAACACCTTTTGG | CCTTCCGTTAGGGTTGAAAA | 3 | 0.5366 | 0.1897 | 0.5120 | 0.5877 | C12:5932858 | 6723804 |
| Hs4260 | GGAACGTGTGATTCATGCTT | TTTCTCTGTCCCCCTTCATC | 3 | 0.3716 | 0.1776 | 0.5877 | 0.6619 | Scaffold00001:  7167108 | 7967487 |
| Hs4209 | CACTCCCTCCCTAGTCCAAA | ATCCAAAGAGCTGCAGGAGT | 3 | 0.8123 | 0.1315 | 0.2911 | 0.3190 | C04:151388 | 1785203 |
| Hs4365 | GGCCCGATATGTTGATGAAT | TCTTGAGCTGAGAAGGCAGA | 2 | 0.5436 | 0.1717 | 0.3731 | 0.4962 | C22:1486322 | 8599974 |
| Hs4082 | AAATGCCAGCACACATTGTC | AAACAATCACCACTTGCTGC | 2 | 0.6495 | 0.1467 | 0.3517 | 0.4553 | C01:6707918 | 8650717 |
| Hs4094 | CAGGGTTGGGTTCTGAGTTT | ATATTTGGAGGCGGTGAAAG | 2 | 0.6223 | 0.1304 | 0.3596 | 0.4701 | C14:4385787 | 8468986 |
| Hs4271 | AGGCCCATAACCATTTCAAG | TGCAAATATCAGGAGTGCCT | 2 | 0.8225 | 0.0949 | 0.2494 | 0.2920 | Scaffold00019:  801040 | 2909052 |
| Hs4325 | GGCATGCAGGACTCTGAAAT | TAAGGCGTCTTTCATCATGG | 5 | 0.3802 | 0.2312 | 0.6656 | 0.7154 | C01:7508382 | 8650717 |
| Hs4398 | AGGGGGAAATTCATTGACAG | TTATTTGGGAGTTTCGAGGG | 4 | 0.6352 | 0.1858 | 0.4551 | 0.5189 | C12:1830385 | 6723804 |
| Mean |  |  | 2.4732 | 0.7216 | 0.1014 | 0.3019 | 0.3655 |  | 5480755 |

Note: ^a^ data behind contig or scaffold number indicates the mapping position of the forward primer sequence in the sesame genome.
