# Supplementary material for: Association Mapping of Seed Oil and Protein Content in Sesamum indicum L. Using SSR Markers
Source: PLoS One. 2014 Aug 25;9(8):e105757. doi: 10.1371/journal.pone.0105757 (PMC4143287; doi:10.1371/journal.pone.0105757)
Supplement: Table S3 — Association mapping of the OC and PC traits using MLM method. (DOCX) [file pone.0105757.s003.docx]

Table S3 Association mapping of the OC and PC traits using MLM method

| **Trait** | **Marker** | **2011** | | | | |  | **2012** | | | | | | | |
| --- | --- | --- | --- | --- | --- | --- | --- | --- | --- | --- | --- | --- | --- | --- | --- |
|  |  | **Pingyu** | |  | **Yuanyang** | |  | **Pingyu** | |  | **Yuanyang** | |  | **Xinyang** | |
|  |  | **F_marker_** | ***P*_marker_** |  | **F_marker_** | ***P*_marker_** |  | **F_marker_** | ***P*_marker_** |  | **F_marker_** | ***P*_marker_** |  | **F_marker_** | ***P*_marker_** |
| OC | Hs235 | 6.61 | 1.50E-03 |  | -- | -- |  | -- | -- |  | 5.54 | 4.30E-03 |  | 9.10 | 1.39E-04 |
|  | Hs345 | 7.78 | 5.10E-06 |  | 10.36 | 5.96E-08 |  | 9.44 | 2.90E-07 |  | 12.06 | 3.30E-09 |  | 9.55 | 2.37E-07 |
|  | Hs376 | 5.29 | 5.40E-03 |  | -- | -- |  | 7.33 | 7.60E-04 |  | 7.23 | 8.36E-04 |  | -- | -- |
|  | Hs4061 | 6.71 | 1.40E-03 |  | 6.52 | 1.70E-03 |  | 6.04 | 2.60E-03 |  | 7.32 | 7.67E-04 |  | 4.76 | 9.10E-03 |
|  | Hs4381 | 9.61 | 8.59E-05 |  | 11.70 | 1.19E-05 |  | 12.78 | 4.32E-06 |  | 12.60 | 5.09E-06 |  | 11.28 | 1.77E-05 |
|  | Hs485 | 5.06 | 1.90E-03 |  | 5.78 | 7.23E-04 |  | 8.00 | 3.56E-05 |  | 6.42 | 3.01E-04 |  | 6.99 | 1.40E-04 |
|  | Hs586 | 11.66 | 1.24E-05 |  | 6.71 | 1.40E-03 |  | 13.91 | 1.51E-06 |  | 10.72 | 3.00E-05 |  | 8.01 | 3.94E-04 |
|  | Hs635 | 5.96 | 2.80E-03 |  | 6.47 | 1.70E-03 |  | 5.69 | 3.70E-03 |  | 6.82 | 1.20E-03 |  | 4.90 | 7.90E-03 |
|  | Hs1036 | 11.88 | 1.03E-05 |  | 11.48 | 1.51E-05 |  | 9.53 | 9.42E-05 |  | 11.97 | 9.51E-06 |  | 7.34 | 7.62E-04 |
| PC | Hs205 | 4.67 | 3.84E-04 |  | -- | -- |  | 4.19 | 1.00E-03 |  | 3.61 | 3.40E-03 |  | -- | -- |
|  | Hs345 | 5.97 | 1.16E-04 |  | 6.36 | 5.96E-05 |  | -- | -- |  | 6.34 | 6.11E-05 |  | 6.67 | 3.49E-05 |
|  | Hs377 | 2.99 | 1.20E-03 |  | -- | -- |  | -- | -- |  | 3.51 | 1.97E-04 |  | 2.67 | 3.80E-03 |
|  | Hs4061 | 7.37 | 7.29E-04 |  | 5.57 | 4.10E-03 |  | 5.56 | 4.20E-03 |  | 6.88 | 1.20E-03 |  | -- | -- |
|  | Hs4381 | 12.62 | 5.03E-06 |  | 8.77 | 1.90E-04 |  | 13.02 | 3.44E-06 |  | 11.08 | 2.13E-05 |  | 17.23 | 7.05E-08 |
|  | Hs485 | 6.05 | 4.98E-04 |  | 5.63 | 8.77E-04 |  | -- | -- |  | 5.55 | 9.83E-04 |  | 5.04 | 2.00E-03 |
|  | Hs672 | 3.55 | 7.30E-03 |  | -- | -- |  | 6.09 | 9.40E-05 |  | 4.94 | 6.88E-04 |  | 4.17 | 2.60E-03 |
|  | Hs1036 | 9.21 | 1.28E-04 |  | -- | -- |  | 5.07 | 6.70E-03 |  | 6.89 | 1.20E-03 |  | 11.79 | 1.13E-05 |
|  | Hs1956 | -- | -- |  | 5.32 | 5.30E-03 |  | -- | -- |  | 4.70 | 9.80E-03 |  | 4.71 | 9.60E-03 |

-- refers to no markers detected under the environment.
