# Supplementary material for: Association Mapping of Seed Oil and Protein Content in Sesamum indicum L. Using SSR Markers
Source: PLoS One. 2014 Aug 25;9(8):e105757. doi: 10.1371/journal.pone.0105757 (PMC4143287; doi:10.1371/journal.pone.0105757)
Supplement: Table S4 — Functions and involved pathways of the candidate genes. (DOCX) [file pone.0105757.s004.docx]

**Table S4 Functions and involved pathways of the candidate genes**

| Candidate gene | **Activity** | **Pathway** |
| --- | --- | --- |
| C01.526 | Lipid Transfer Protein | Suberin III  Fatty Acid Elong & Wax |
| C01.548 | Acyl-ACP Thioesterase A | Prok Gal/Sulpho I  Fatty Acid Synth  Plast FAE & Desat |
| C01.575 | Lipid Acylhydrolase-like | Oxylipin I  Oxylipin II |
| C01.601 | Acyl-CoA Thioesterase | FA Elong & Wax  TAG Degradation |
| C01.873 | ABC Transporter | Cutin I & II  Suberin III  FA Elong & Wax |
| C01.883 | ABC Transporter | Cutin I & II  Suberin III  Fatty Acid Elong & Wax |
| C01.928 | Diacylglycerol Acyl transferase | TAG Synthesis |
| C02.739 | Midchain Alkane Hydroxylase | Fatty Acid Elong & Wax |
| C04.38 | ABC Transporter | Cutin I & II  Suberin III  Fatty Acid Elong & Wax |
| C04.56 | Phosphatidylinositol-Phosphate Kinase type III | Signaling |
| C04.81 | Phosphatidylinositol-Phosphate Kinase type IB | Signaling |
| C04.96 | Phosphatidylinositol-Phosphate Kinase type IB |  |
| C04.767 | Phosphoinositide 5-Phosphatase Type II | Signaling |
| C04.786 | Stearoyl-ACP Desaturase | Plast FAE & Desat  Fatty Acid Synth  Prok Gal/Sulpho I |
| C13.388 | Phosphatidylinositol-Phosphate Kinase type IB | Signaling |
| C13.471 | Hydroxyacyl-ACP Dehydrase | Fatty Acid Synth  Mito FAS & Lipoic |
| C13.504 | ABC Transporter | Cutin I & II  Suberin III  Fatty Acid Elong & Wax |
| C13.514 | Subunit of Serine Palmitoyltransferase | Sphingolipid I |
| C14.49 | Lipid Transfer Protein | Suberin III  FA Elong & Wax |
| C14.66 | Long-Chain Acyl-CoA Synthetase | Fatty Acid Synth  Plast FAE & Desat  Prok Gal/Sulpho I |
| C14.111 | Sphingobase-D8 Desaturase | Sphingolipid I |
| C14.132 | Diacylglycerol Kinase | Signaling |
| C14.359 | CDP-DAG Synthase | Mito LipoPolySach  Prok Gal/Sulpho I  Euk Phospholipid |
| C14.413 | Lipid Transfer Protein | FA Elong & Wax |
| C14.428 | Acylhydrolase (DAD1-like) | Oxylipin I & II |
| C15.92 | Linoleate Desaturase | TAG Synthesis  Euk Phospholipid |
| C25.69 | Lipoate Synthase | Fatty Acid Synth  Mito FAS & Lipoic |
| C26.417 | Phosphatidylserine Synthase | Euk Phospholipid |
| C26.454 | Phosphatidylinositol-Phosphate Kinase type III | Signaling |
| C26.515 | Acyl CoA Binding Protein | Euk Phospholipid |
| sf00001.95 | Dihydrosphingosine Delta-4 Desaturase | Sphingolipid I |
| sf00044.12 | Phosphatidic Acid-Binding Protein | Trafficking  Euk Gal/Sulpho |
| sf00044.41 | SWI / SWF nuclear-localized chromatin  remodeling factor of the CHD3 group | TAG Synthesis |
| sf00044.61 | Acyl acceptor Acyl transferase | Euk Gal/Sulpho  TAG Synthesis  Euk Phospholipid |
| sf00044.90 | Translocase | -- |
| sf00044.113 | Choline Kinase | Euk Phospholipid  TAG Synthesis |
